# Pro-inflammatory secreted virulence factors of *Staphylococcus capitis* causing a rare occurrence of severe native hip joint infection

**DOI:** 10.1128/asmcr.00067-24

**Published:** 2025-02-11

**Authors:** Patrick M. Schlievert, Samuel H. Kilgore, Bradley Ford, Donald Y. M. Leung, Poorani Sekar

**Affiliations:** 1 Department of Microbiology and Immunology, Carver College of Medicine, The University of Iowa 311821 , Iowa City, Iowa, USA; 2 Internal Medicine, Carver College of Medicine, The University of Iowa 194453 , Iowa City, Iowa, USA; 3 Department of Pathology, Carver College of Medicine, The University of Iowa 160416 , Iowa City, Iowa, USA; 4 Department of Pediatrics, National Jewish Health 2930 , Denver, Colorado, USA; Pattern Bioscience, Austin, Texas, USA

**Keywords:** *Staphylococcus capitis*, abscess, osteomyelitis, inflammation, polysaccharide intercellular adhesin, autolysin, immunodominant antigen B

## Abstract

**Background:**

*Staphylococcus capitis* occasionally causes human infections. We report a case of *S. capitis* infection associated with inflammatory destructive hip abscess/osteomyelitis.

**Case Summary:**

A 63-year-old man with severe hip osteoarthritis and recent left hip replacement presented with 2 months of worsening right hip pain. Radiographic imaging showed right femoral head destruction. The patient had no known hip trauma. His white blood cell count, C-reactive protein, and erythrocyte sedimentation rate were abnormal. Pre-operative aspirate of the hip showed purulent fluid. The examining radiologist believed the changes seen were due to inflammatory arthritis with infection from low-virulence organisms. The patient underwent image-guided biopsies of the synovium and the acetabulum, which showed pure cultures of *S. capitis* and infiltrating neutrophils. The patient’s native hip was removed, and an antibiotic spacer was placed. The patient was treated with intravenous cefazolin (6 weeks), after which he was transitioned to oral cefadroxil. The patient is doing well. The *S. capitis* was analyzed by nucleotide sequencing and biochemically for secreted virulence factors. The strain contained the polysaccharide intercellular adhesin biofilm operon. The strain was negative for urease, hemolysins, and major superantigens. The organism secreted a neutrophil pro-inflammatory autolysin protease and an analog of the immunodominant antigen B of *S. aureus*, previously with no known biological function.

**Conclusion:**

Our data suggest that the inflammatory lesion in the patient’s right hip was due to *S. capitis* subspecies *capitis*, and furthermore, it is likely that two secreted proteins (autolysin and an analog of immunodominant antigen B) contributed to the inflammation.

## INTRODUCTION

Staphylococci are Gram-negative, catalase-positive cocci, further subdivided as coagulase positive and coagulase negative. Coagulase-negative staphylococci (CoNS) are typically considered opportunistic human pathogens, originating from skin and mucous membranes (1, 2). From 40% to 60% of humans are colonized with the CoNS, *S. capitis* (3).

*S. capitis* occasionally are associated with human infections (4). These organisms cause late-onset sepsis infections in low birth weight infants and may cause infections in older persons, including osteomyelitis and surgical implant infections. *S. capitis* strains lack many of the typical toxin virulence factors of *S. aureus* but often have the polysaccharide intercellular adhesin (*ica*) operon, important in biofilm formation (4). *S. capitis* is sub-speciated into *urealyticus* because of urease production and *capitis*, which lack urease (4).

This study addresses an adult patient with an inflammatory and destructive right hip abscess plus osteomyelitis, due to *S. capitis* subspecies *capitis*, and characterizes novel secreted virulence factors that may contribute to the inflammation. The destruction in the right hip joint in our case over a short time period is instructive, as it is not commonly seen in clinical practice.

## CASE PRESENTATION

**Fig 1:**
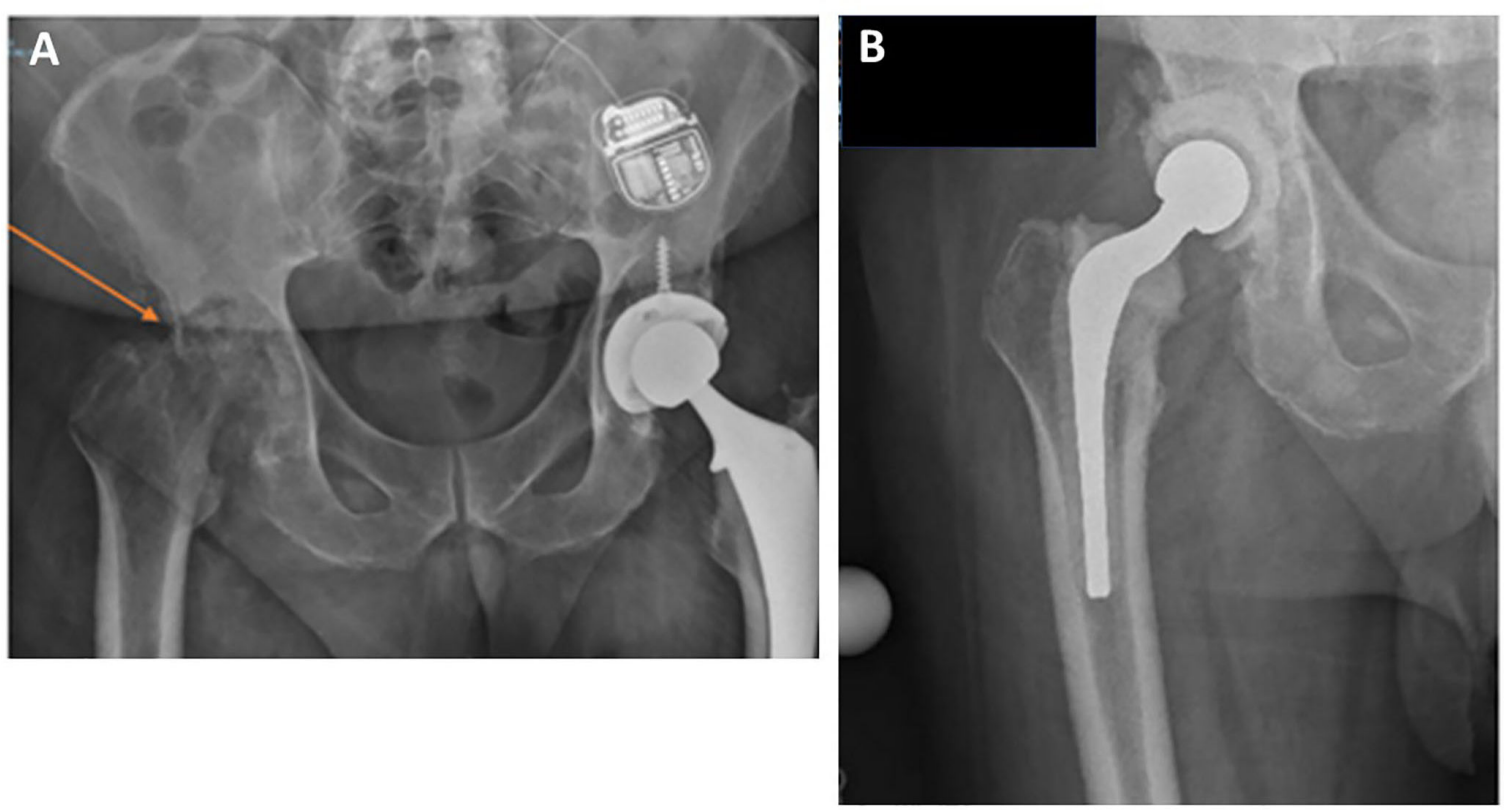
(**A**) X-ray image of the right hip of a patient with destructive changes of his hip (see arrow) and (**B**) X-ray image of the right hip of the patient post-surgery done to replace his native hip with an antibiotic spacer.

A 63-year-old man with a history of hypertension, benign prostatic hypertrophy, and severe osteoarthritis of both hips, and with recent left hip replacement (2 months prior), presented with progressively worsening right hip pain. X-ray of the pelvis 2 months ago indicated left hip arthroplasty and severe osteoarthritic changes of the right hip joint. The patient continued to have right hip pain, which worsened over only 2 months, with imaging showing acute worsening, with a destroyed femoral head (Fig. 1A). Upon presentation, the patient denied redness, warmth, drainage at his right hip, fevers, chills, sweats, or weight loss. The patient did not have known trauma to his hip, and he did not have injections to the right hip.

His white blood cell count on admission was 25,300 /µL (reference range 4,000–11,500/µL), C-reactive protein was 17.3 mg/dL (reference range <0.5 mg/dL), and erythrocyte sedimentation rate was 82 mm/h (reference value 20 mm/h). Pre-operative aspirate of the right hip showed purulent fluid. There were adhesions under the fascia, and necrotic and friable tissue, which also indicated inflammation. The samples taken showed many polymorphonuclear leukocytes (PMNs) microscopically.

On differential diagnosis, the examining radiologist believed that the bony changes seen in the patient could be due to inflammatory arthritis, with infection due to low-virulence organisms. A nuclear medicine bone scan was performed, which showed an increased uptake of indium at the right hip and femur, extending to the mid-femur, creating concern for osteomyelitis. The patient underwent image-guided biopsies of the synovium and acetabulum, which showed pure cultures of *S. capitis* and many PMNs by microscopy. Four sets of blood cultures (Virtuo system, bioMerieux; each set consisted of paired FA PLUS and FN PLUS bottles) were done on consecutive days, and all were negative. The *S. capitis* was definitively identified by matrix-assisted laser desorption ionization–time of flight (MALDI-TOF) mass spectrometry (Bruker Biotyper Sirius instrument, research use database version 12–11897; with scores ranging from 2.03 to 2.21) by our Diagnostic Microbiology Facility. There were no patient features to suggest an underlying predisposition to invasive *S. capitis* infection. HIV screen was negative, but he was obese (body mass index: 34). *S. capitis* was thus considered the cause of the patient’s presentation. The patient was taken to the operating room, his native right hip was resected, and an antibiotic spacer was placed (Fig. 1B).

Five sets of deep, sterile tissue cultures were taken from various parts of the hip, with four of five samples growing pure *S. capitis*. Image-guided biopsies were obtained on initial admission, and surgical cultures 1 week later. Thus, these were obtained in a very short interval, leading us to believe that there were no isolate changes over this time period. The *S. capitis* was oxacillin-susceptible according to both cefoxitin screening and oxacillin MIC testing on the Vitek II system. The oxacillin MIC was ≤0.25 µg/mL, and thus, additional direct testing for *mecA* was not performed (per Clinical & Laboratory Standards Institute M100). This indicated the organism did not harbor *mecA*-mediated beta-lactam resistance. Hence, the patient was treated with intravenous cefazolin (2 g every 8 h) for 6 weeks, after which he was transitioned to oral cefadroxil (500 mg by mouth twice daily). The reason cefadroxil was chosen was because of a longer half-life with a dosing frequency of twice daily, to which patients are better able to adhere than the 4 times/day dosing frequency for cephalexin. The patient is now doing well with the pain resolving and patient again being mobile. He has decided to keep the spacer as his permanent hip unless he has mechanical issues later.

Genomic DNA from the patient’s *S. capitis* was submitted to the SeqCenter, Pittsburgh, PA, for nucleotide sequencing. Three sequences were obtained, one being the 2,476,100 base-pair genome and two presumed to be extra-chromosomal DNA (plasmids or bacteriophages) of 19,048 and 40,697 base pairs. When examined for known staphylococcal virulence operons, the isolate contained the polysaccharide intercellular adhesin (*ica*) biofilm operon (5, 6). By genome examination and PCR, the isolate lacked genes for the known highly inflammatory superantigens (7–9). This was not surprising since the patient did not have evidence of high fever, hypotension, and multi-organ changes, commonly associated with toxic shock syndrome (10–12).

The strain was not hemolytic (13). There was no urease gene. The *S. capitis* strain was urease negative as determined by biochemical assay, whereas the positive control *Proteus mirabilis* strain was urease positive. Thus, this organism was classified as *S. capitis* subspecies *capitis* (4). The genome lacked the pro-inflammatory methionine-rich protein of *S. aureus* (14). The strain was negative for staphylococcal lipase activity (15), whereas purified lipase was positive. By biochemical assay (Thermoscientific Pierce Colorimetric Assay), culture fluids from the *S. capitis* and positive control *S. aureus* MN8 were positive for proteases. From these collective studies, the strain contained intercellular polysaccharide adhesin and secreted protease virulence factors but lacked other known secreted *S. aureus* virulence factors (8, 9, 16).

**Fig 2:**
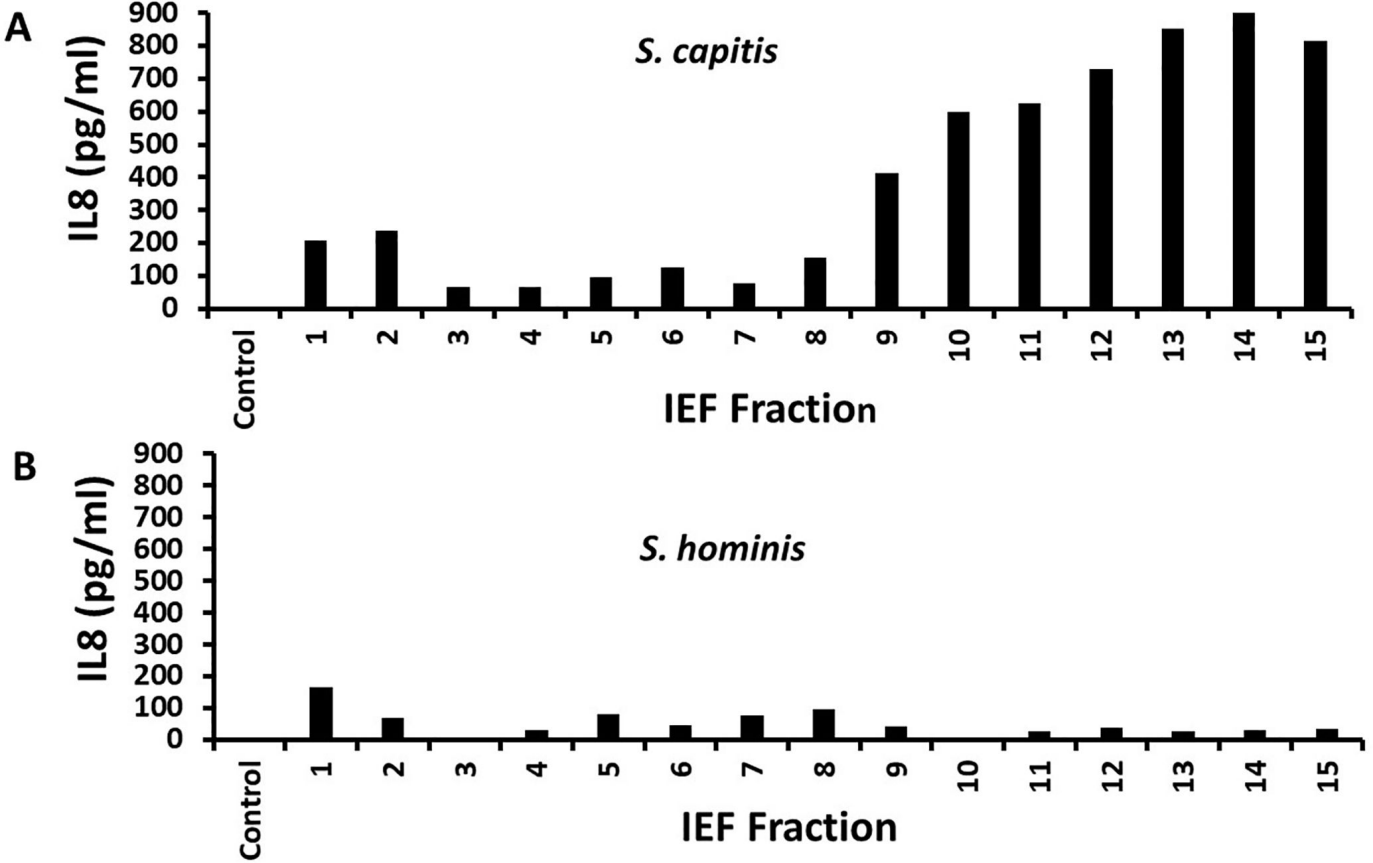
(**A**) IL8 production by HVECs in a 6 h assay in response to 15 one-centimeter fractions from isoelectric focusing of secreted proteins from *S. capitis*, and (**B**) IL8 production by HVECs in a 6 h assay in response to 15 one-centimeter fractions from isoelectric focusing of secreted proteins from *S. hominis*. Controls were HVECs without fractions added.

We next tested if the strain produced novel secreted pro-inflammatory virulence factors. We prepared cell-free culture fluids of the *S. capitis* strain after aerobic growth in Todd Hewitt broth, followed by our standard methods (17) for purification of secreted toxins: ethanol precipitation and preparative thin-layer isoelectric focusing (pH gradient: 3.5–10). We harvested 15 one-centimeter fractions from the isoelectric focusing plate. These were tested (18) for the stimulation of human vaginal epithelial cells (HVECs) to produce pro-inflammatory interleukin 8 (IL8), as a measure of PMN-induced inflammatory cytokines (Fig. 2A). IL8 is a known chemokine that specifically attracts PMNs to infection sites. Fractions 9–15 caused the greatest production of IL8 from HVECs, 400–900 pg/mL in our standard 6 h assay. As an additional control, we performed the same analysis on a recent clinical isolate of *Staphylococcus hominis* and did not observe IL8 production in the fractions, including fractions 9–15 (Fig. 2B). If the IL8 data from the pooled *S. capitis* 9–14 fractions were compared with the same fractions of *S. hominis*, the means were significantly different with *P* < 0.001 by Student’s *t* test.

**Fig 3:**
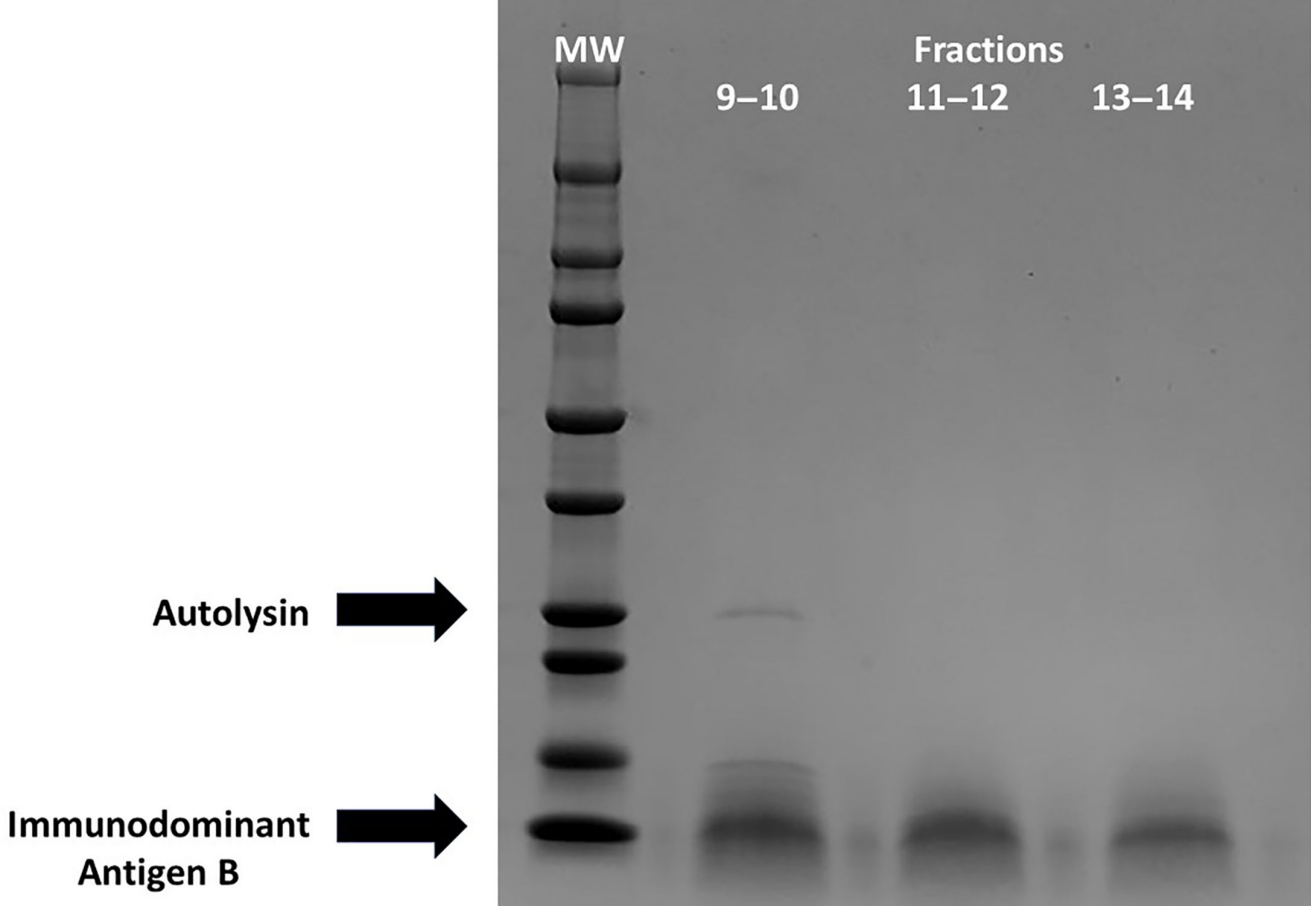
Sodium dodecyl sulfate polyacrylamide gel electrophoresis of fractions from 3.5 to 10 isoelectric focusing of *S. capitis* secreted proteins. MW = molecular wt markers; pooled fractions 9–10, 11–12, and 13–14. Arrows indicate protein bands analyzed by mass spectrometry.

The 9–10, 11–12, and 13–14 fractions from *S. capitis* were individually pooled and tested for protein content with use of the Bio-Rad reagent. Fraction 15 contained insoluble material; hence, this fraction was omitted from the analysis. The pooled samples were matched for protein content and subjected to SDS-PAGE (Fig. 3) (MiniPROTEAN TGX Gels, 4%–20%, Bio-Rad) (19). Proteins of molecular weights 25,000 and 17,000 were detected. When evaluated by MALDI-TOF mass spectrometry, the upper band belonged to the autolysin group of proteases. Autolysins play a critical role in bacterial cell division by combinations of protease and glucanase activity. The lower dominant band was identified as related to the *S. aureus* immunodominant antigen B (20). This protein is considered bacterial cell wall-associated (20), but we isolated a secreted form. The data suggest that at least the immunodominant antigen B, but possibly both proteins stimulated IL8 production by HVECs. The extra band in fraction 9–10 disappeared after lyophilization followed by re-solubilization in water. This band could have been a breakdown product of the autolysin or an unstable protein.

## DISCUSSION

Our manuscript describes a patient with right hip destruction associated with suspected PMN-induced inflammation caused by a strain of *S. capitis* subspecies *capitis*. The hip destruction occurred over the course of only 2 months, more rapidly than would be expected from this organism. In order to investigate the possible mechanism underlying this rapid infection progression, we characterized the *S. capitis* genome and culture fluids for the production of secreted virulence factors. Three possible factors were identified: polysaccharide intercellular adhesion, autolysin protease, and immunodominant antigen B. The immunodominant antigen B of *S. aureus* has been previously identified as a strongly immunogenic, nucleic acid binding protein, but with no role in biofilm formation, and unclear function in virulence (20).

The hip joint of this patient could be viewed as a biofilm (tissue adherent) infection as opposed to a planktonic (liquid non-adherent) infection. Such an infection would be expected to yield very high numbers of *S. capitis*, partially protected from the host immune system (21), leading subsequently to high inflammation and ultimately hip destruction. The polysaccharide intercellular adhesin is likely to have contributed to biofilm formation (4, 5). Biofilm formation requires bacterial cells first to adhere to a substrate, such as the patient’s hip, followed by intercellular adhesion, facilitated by the polysaccharide intercellular adhesin, proteins, and extracellular nucleic acids (collectively referred to as extracellular polymeric substances) as reviewed in (6, 22).

Evaluation of the patient and his right hip indicated PMN infiltration into the hip site. This was suggested by the PMN infiltrates into the infection site, adhesions under the fascia, necrotic and friable tissue, and elevated systemic numbers of white blood cells and C-reactive protein. Because of these findings, we evaluated the causative agent, *S. capitis*, for secreted virulence factors that could contribute to the destructive inflammatory processes. We identified two proteins (autolysin protease and immunodominant antigen B) that induced significant IL8 production by HVECs, noting that epithelial cells are often the first cell types to interact with pathogens. We have published what we refer to as “outside in” signaling for microbes to cause human infections (23, 24). Briefly, this signaling mechanism refers to microbial products interacting with human cells, causing IL8 (and other chemokines) production, with consequent PMN influx and harmful inflammation. The immunodominant antigen B of *S. capitis* is so-named because it induces antibody responses in animals, but until our study, it had no known role in virulence. Our studies showed this protein can be secreted, and when secreted, it can induce significant IL8 production by epithelial cells. We note that there is no simple way to extrapolate IL8 production *in vitro*, as we have shown in our studies, to the human setting of *S. capitis* infection. However, we presume that any amount of IL8 produced *in vivo* would initiate PMN attraction through IL8 receptors.

### Conclusion

Our data suggest that the inflammatory lesion in the patient’s right hip was due to *S. capitis* subspecies *capitis*, and furthermore, it is likely that the two secreted pro-inflammatory proteins (autolysin and immunodominant antigen B) contributed to the inflammation. No other secreted products were identified from the strain.

## Data Availability

The genome sequence of our *S. capitis* subspecies *capitis* strain has been uploaded to the NCBI website with accession number JBISAR000000000.
